# The current role of elbow hemiarthroplasty compared to total arthroplasty in the treatment of distal humerus fractures in the elderly. Twice the trouble or half the hassle?

**DOI:** 10.1016/j.xrrt.2025.06.015

**Published:** 2025-07-10

**Authors:** Felix Peuker, Niels M. van der Hoeven, Roland S. Camenzind, Robert-Jan Derksen, Frank J.P. Beeres, Bryan J.M. van de Wall, NEXT study group

**Affiliations:** a Department of Orthopedic and Trauma Surgery, Cantonal Hospital Lucerne, Lucerne, Switzerland; b Department of Health Sciences and Medicine, University of Lucerne, Lucerne, Switzerland; c Department of Trauma Surgery, University Medical Center Utrecht, Utrecht, the Netherlands; d Department of Trauma Surgery, Leiden University Medical Center, Leiden, the Netherlands; e Department of Trauma Surgery, Zaans Medical Center, Zaandam, the Netherlands

**Keywords:** Distal humerus fractures, Elderly, Trauma surgery, Elbow arthroplasty, Octogenarians, Functional outcomes

## Abstract

**Background:**

Unreconstructable distal humerus fractures in elderly patients are commonly treated with total elbow arthroplasty (TEA). However, TEA has the major disadvantage of lifelong weight-bearing restrictions. Elbow hemiarthroplasty (EHA) is an alternative that allows full weight-bearing, but it is less commonly performed, and there's a lack of high-quality original research directly comparing it to TEA. Therefore, a systematic review and meta-analysis of all available research were conducted to better understand the differences.

**Methods:**

A systematic review and meta-analysis were conducted following the Preferred Reporting Items for Systematic Reviews and Meta-Analyses guidelines. Studies on EHA or TEA with at least three months of follow-up were included. Primary outcome was the Mayo Elbow Performance Score (MEPS). Secondary outcomes included the Disabilities of the Arm, Shoulder, and Hand (DASH) score, revision rates, implant-related and general postoperative complications, and range of motion.

**Results:**

Thirty-six studies were included, with 502 TEA and 192 EHA patients. MEPS scores were similar (TEA: 87.6; EHA: 85.9; *P* = .505), but EHA had a significantly better DASH score (19.6 vs. 40.9; *P* < .001). TEA and EHA had equivalent implant-related (TEA: 11.1, EHA: 7.6%; *P* = .070) and general postoperative complications (TEA: 9.5%, EHA: 11.6%; *P* = .145). Revision rates after implant-related complications were also similar (TEA: 6.4%, EHA: 3.3%; *P* = .051).

**Conclusion:**

TEA and EHA demonstrate comparable MEPS outcomes, with EHA achieving better DASH scores. Complication and revision rates remain similar between the two. These findings suggest that EHA is similar to TEA in treating elderly patients with unreconstructable distal humerus fractures, with the added advantage of no weight-bearing restrictions which could explain the difference in DASH scores.

Distal humerus fractures account for 1% of all extremity fractures. They are particularly challenging to treat, especially in patients over 65 years, where osteoporotic bone contributes to a complex, often intra-articular fracture pattern and reduces anchorage of screws when osteosynthesis is performed.^8^^,^^13^^,^^43^ Although open reduction and internal fixation is the standard treatment, it is not always possible due to the previously described constraints. These patients might profit from joint replacement using either total elbow arthroplasty (TEA) or elbow hemiarthroplasty (EHA).^49^ Historically, TEA has been the primary choice, as orthopedic surgeons typically have significantly more experience with this implant.^26^ However, a major drawback—although generally considered more acceptable in elderly patients—is the need for lifelong weight-bearing restrictions due to the risk of implant loosening, early wear of the bushing elements, and the subsequent need for revision surgery.^9^ In contrast, replacing only the distal humerus while preserving the proximal ulna with an EHA generally eliminates these restrictions.

High-quality evidence comparing EHA to TEA is limited, with most existing literature consisting of case series.^9^^,^^45^ A few systematic reviews have been published on this subject; however, all were published without including the recent randomized controlled trial (RCT) by Jonsson et al, which could shed new light on the matter.^25^ Traditionally, case series are not suitable for inclusion in meta-analyses due to the high risk of confounding. This confounding arises because treatment choices are often based on patient risk profiles, leading to differences in study population characteristics across case series. However, as previously noted, the decision to use TEA or EHA is typically driven by surgeon preference and expertise rather than the patient risk profile.^22^ This theoretically reduces the risk of confounding when including case series in a meta-analysis on this specific topic. Emerging evidence supports this assumption.^6^^,^^7^^,^^17^ Therefore, a systematic review and meta-analysis were conducted, incorporating not only comparative studies but also case series, to compare TEA and EHA in terms of functional outcomes, complications, and revision rates. In addition, the assumption that treatment groups would have comparable study populations was internally validated, supporting the chosen unconventional meta-analytical approach.

## Methods

### Study design

This systematic review and proportional meta-analysis was conducted in accordance with the Preferred Reporting Items for Systematic Reviews and Meta-Analyses guidelines.^38^ Ethical approval was not required for this study.

### Search strategy

A comprehensive literature search was performed in the PubMed, Embase, Ovid Medline, and CINAHL databases on April 13, 2025. The detailed search strategy, including Medical Subject Headings terms and their synonyms, is provided in Supplementary S1.

### Eligibility criteria

Studies investigating either EHA or TEA for the management of distal humeral fractures were included. The indication for surgery had to be traumatic in nature. In cases where studies reported multiple indications, data had to be separable to isolate the traumatic cases. Exclusion criteria encompassed case reports, technical notes, biomechanical studies, registry studies, systematic reviews, and meta-analyses. Articles lacking full-text availability or not published in English, Dutch, German, or French were excluded. Furthermore, studies were required to report at least one functional outcome measure, range of motion (ROM), or complication rates. A minimum follow-up of three months was necessary, and studies had to include at least five patients with a mean age of 65 years or older.

### Study selection

Two independent reviewers (F.P. and N.H.) screened the titles and abstracts to determine the study eligibility. Full-text versions of potentially eligible studies were further evaluated based on the inclusion criteria, and any excluded study was documented with justification. Discrepancies were resolved through discussion, and if consensus could not be reached, a third reviewer (B.W.) was consulted. In addition, reference lists of review articles and included studies were screened for relevant publications not identified through the initial search. Duplicate records were removed using Rayyan software (Rayyan QCRI; Qatar Computing Research Institute, Ar-Rayyan, Qatar).

### Data extraction

Relevant data were extracted and systematically recorded in a structured spreadsheet (Microsoft Excel; Microsoft Corp., Redmond, WA, USA). The following variables were collected: author names, article title, journal, year of publication, institutional affiliation, country of origin, continent, language, inclusion period, mean follow-up duration, minimum follow-up duration, total number of patients, sex distribution, mean age, study design, surgical strategy, fracture classification (AO/OTA [Arbeitsgemeinschaft für Osteosynthesefragen/Orthopaedic Trauma Association] fracture classification), mean time to surgery, trauma mechanism, implant type, surgical approach, and postoperative protocols.^37^

### Quality assessment

The methodological quality of case series was assessed using the Joanna Briggs Institute Critical Appraisal Tool. Nonrandomized studies were evaluated using the methodological index for non-randomized studies (MINORS) tool, whereas RCTs were assessed using the Cochrane Risk of Bias tool.^40^^,^^54^^,^^58^ Details regarding the methodological quality of the included studies are presented in Supplementary S2-S4.

### Study outcomes

The primary outcome measure was postoperative functional performance of the elbow, assessed using the Mayo Elbow Performance Score (MEPS) at final the follow-up.^39^

Secondary outcomes included the Disabilities of the Arm, Shoulder, and Hand (DASH) score at final the follow-up and the revision and complication rates, occurring up to the final follow-up moment.^23^ A distinction was made between implant-related complications (ie, periprosthetic fracture, mechanical failure, aseptic loosening, dislocation, wear, epicondyle nonunion, and prominent hardware) and general postoperative complications (ie, deep/superficial infection, septic loosening, wound dehiscence, heterotopic ossification, joint stiffness, joint instability, and ulnar neuropathy). All complications were further subcategorized into either requiring reintervention or not. Other secondary outcomes included the final ROM of the elbow, specifically flexion, extension, pronation, and supination, reported in degrees.

### Sensitivity analysis

A sensitivity analysis for study quality was performed on the primary outcome, MEPS. The studies reporting on the MEPS were ranked and categorized into high- and low-quality groups based on the quality assessments, using the 50th percentile as the cutoff. The ranking of the studies and their subsequent allocation into a low-quality group and a high-quality group is further detailed in Supplementary S5-S7.

### Statistical analysis

Continuous variables were presented as means with 95% confidence intervals (95% CIs), or converted accordingly using methods described in the Cochrane Handbook for Systematic Reviews of Interventions.^20^ Dichotomous variables were expressed as counts and proportions. Effect estimates were reported as weighted proportions or means, and relative risk with corresponding 95% CIs was calculated for complication risk analysis. Complications were documented as the number of patients experiencing a specific complication. Given the proportional meta-analysis approach, a statistically significant difference between the hemiarthroplasty and TEA groups was defined by nonoverlapping 95% CIs. Heterogeneity among studies within each treatment group was assessed using the I^2^ statistic. A heterogeneity level exceeding 50% was considered substantial. All statistical analyses were conducted using Stata Statistical Software (version 18; StataCorp, College Station, TX, USA).^56^

## Results

**Figure 1:**
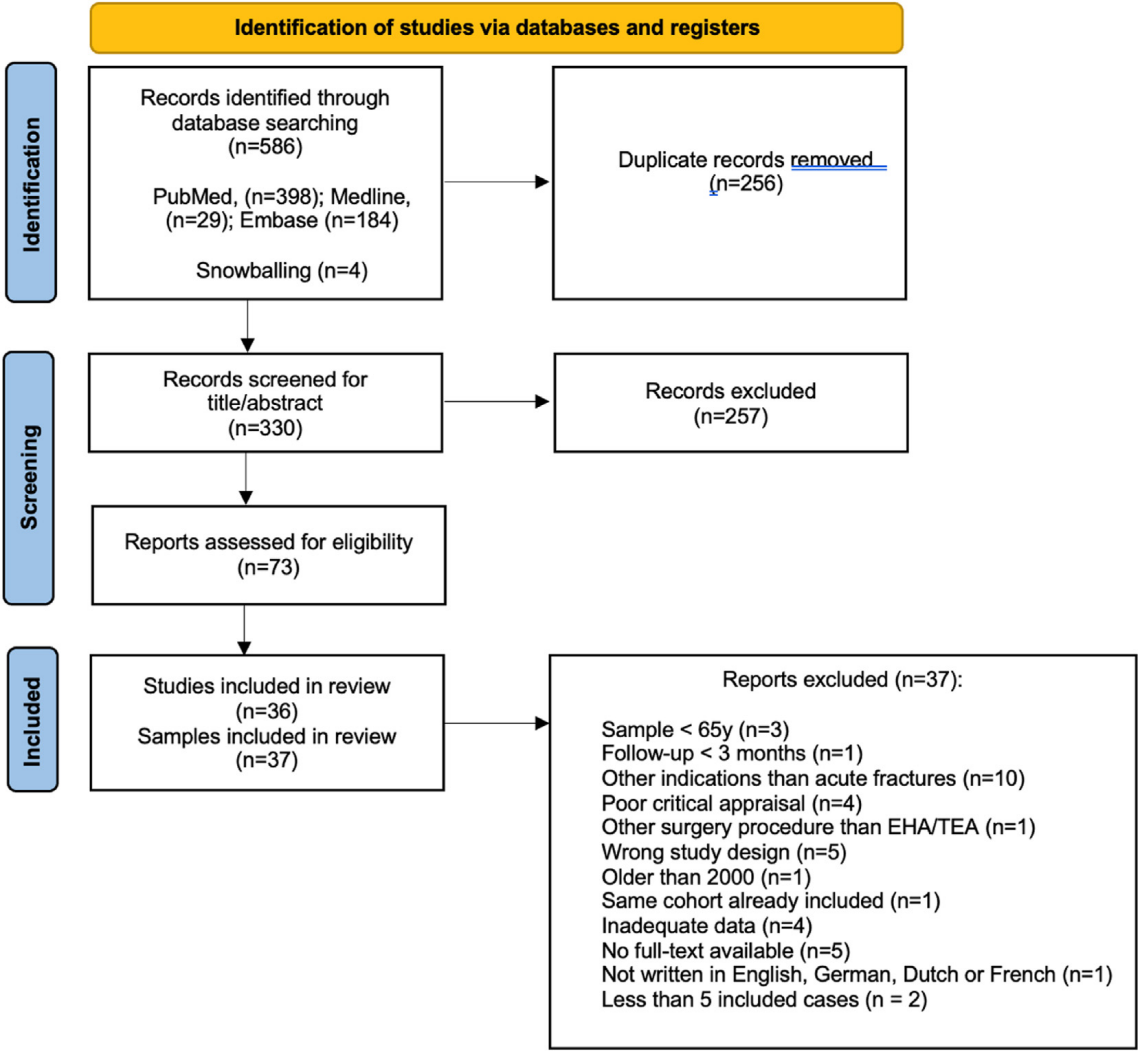
Flowchart of included studies. *EHA*, elbow hemiarthroplasty; *TEA*, total elbow arthroplasty.

The search strategy identified a total of 586 studies, of which 330 were screened based on title and abstract. Following a full-text eligibility assessment, 36 studies, representing 37 distinct samples, were included in the final analysis. The study selection process is illustrated in the Preferred Reporting Items for Systematic Reviews and Meta-Analyses flow diagram (Fig. 1).

### Baseline characteristics

In the TEA group, 36 studies comprising 502 patients were analyzed. The mean age of this group was 75.4 years (95% CI: 73.9-76.9), with a mean follow-up duration of 45.7 months (95% CI: 31.5-59.8). The EHA group included 11 studies, consisting of 192 patients with a mean age of 73.0 years (95% CI: 17.5-75.4) and a mean follow-up of 34.4 months (95% CI: 23.4-45.3). Most fractures were type C (86.8% in the TEA vs. 84.1% in the EHA group), followed by type B (6.8% vs. 15.9%). In the EHA group, the Tornier Latitude (95.7%) was predominantly used and in the TEA group, mostly the Coonrad-Morrey implant (70.9%). The detailed characteristics of all included studies are presented in Tables I and II.

**Table I:** Baseline characteristics for articles reporting on total elbow arthroplasty.

Study	Patients, n	Mean follow-up, mo	Minimum follow-up, mo	Mean age, yr	Design	Fracture type (AO/OTA)	Days from injury to surgery	Implants used
Pooley^47^	11	42 (12)	24	76 (64-91)	Case-series	C (n = 11)	NR	Discovery (n = 4), IBP (n = 7)
Jonsson^25^	17	NR	24	76.9 (7.6)	RCT	B3 (n = 4) C1 (n = 13)	8.1 (4.1)	Latitude (n = 17), Discovery (n = 1)
Schiavi^52^	12	91.2 (15)	60	74.7 (7.6)	Case-series	C (n = 12)	NR	NR
Kim^27^	9	29 (13.3)	12	72.7 (63-85)	Case-series	A2 (n = 1) A3 (n = 1) C3 (n = 7)	27.8 (5-85)	Coonrad-Morrey (n = 9)
Zhang^61^	13	91.5 (19.5)	52	69.3 (6.4)	Cohort study	C (n = 13)	NR	Coonrad-Morrey (n = 13)
Chalidis^12^	11	25 (12)	12	79.6 (75-86)	Case-series	C2 (n = 3) C3 (n = 8)	4.3 (2-8)	Discovery (n = 11)
Liu^32^	22	59.4	NR	71.6 (48.9-88.1)	Case-series	A (n = 1) B (n = 4) C (n = 17)	9	Coonrad-Morrey (n = 2), Discovery (n = 19), Latitude (n = 1)
Logli^33^	22	52.8 (40.2)	24	74 (50-90)	Cohort study	A (n = 1) B (n = 6) C (n = 15)	NR	Coonrad-Morrey (n = 8), Discovery (n = 4), Latitude (n = 3), Nexel (n = 7)
Lopiz^34^	11	67 (5.5)	60	82 (80-88)	Cohort study	C1 (n = 2) C2 (n = 2) C3 (n = 7)	11 (5-14)	Coonrad-Morrey (n = 9)
Sørensen^55^	20	21 (12)	4	77 (55-95)	Case-series	A2 (n = 2) B3 (n = 1) C3 (n = 17)	9.1 (1-22)	Coonrad-Morrey (n = 20)
Robinson^50^	26	37.8 (14.5)	12	70 (42-90)	Cohort study	A3 (n = 2) B3 (n = 1) C1 (n = 21) C3 (n = 2)	NR	Coonrad-Morrey (n = 26)
Pogliacomi^46^	20	60 (36)	24	74.1 (68-86)	Case-series	C3 (n = 20)	NR	Coonrad-Morrey (n = 12), Latitude (n = 8)
Prasad^48^	19	156 (22.5)	120	68 (40-80)	Case-series	A3 (n = 4) B1 (n = 2) B3 (n = 3) C1 (n = 4) C3 (n = 6)	NR	Coonrad-Morrey (n = 19)
Macknet^35^	69	12.6 (6.5)	3.6	73.6	Cohort study	A (n = 9) B (n = 3) C (n = 56) Unknown (n = 1)	NR	
Antuña^4^	16	57 (16.8)	24	76 (57-89)	Case-series	B3 (n = 2) C2 (n = 2) C3 (n = 12)	8 (2-45)	Coonrad-Morrey (n = 14)
Dehghan^14^	25	92.4 (41.1)	12	78	RCT	C (n = 25)	NR	Coonrad-Morrey (n = 25)
Baik^5^	43	34 (27.5)	6	79 (65-91)	Cohort study	C1 (n = 10) C2 (n = 10) C3 (n = 23)	NR	Coonrad-Morrey (n = 43)
Ali^3^	20	63.2 (18)	36	72 (62-92)	Case-series	A3 (n = 1) B2 (n = 1) B3 (n = 4) C3 (n = 14)	10 (1-28)	Coonrad-Morrey (n = 20)
Linn^31^	7	43 (33.5)	4	74 (56-86)	Case-series	C (n = 7)	6 (2-19)	Coonrad-Morrey (n = 7)
Lami^29^	21	38.4 (15)	24	81.3 (70-92)	Case-series	A3 (n = 2) C (n = 19)	9 (2-22)	Coonrad-Morrey (n = 21)
Giannicola^19^	10	34.1 (3.3)	30	78 (66-89)	Case-series	C2 (n = 2) C3 (n = 8)	3 (2-5)	Discovery (n = 10)
Ducrot^16^	20	43.2 (12.8)	20.4	80 (65-93)	Case-series	A (n = 2) B (n = 2) C (n = 15) Unknown (n = 1)	8 (1-45)	Coonrad-Morrey (n = 20)
Lee^30^	7	24.9 (3.8)	17	72.9 (55-85)	Case-series	A (n = 4) B (n = 1) C2 (n = 2)	37.3 (2-104)	Coonrad-Morrey (n = 7)
McKee^36^	25	24	NR	78	RCT	C1 (n = 2) C2 (n = 6) C3 (n = 17)	NR	Coonrad-Morrey (n = 25)
Kraus^28^	12	28 (17)	7	81 (10)	Case-series	C3 (n = 12)	NR	Coonrad-Morrey (n = 12)
Frankle^18^	12	45 (19)	3	72 (65-88)	Cohort study	C2 (n = 8) C3 (n = 4)	7.8 (2-30)	Coonrad-Morrey (n = 12)
Weighted	**502**∗	**45.7 (31.5-59.8)**	**20.1 (10.3-29.9)**	**75.4 (73.9-76.9)**	**Case series (47.4%)** **Cohort study (39.2%)** **RCT (13.4%)**	**A (6.0%)** **B (6.8%)** **C (86.8%)**	**4.1 (2.5-5.7)**	**Discovery (13.8%)** **IBP (2.5%)** **Latitude (10.3%)** **Coonrad-Morrey (70.9%)** **Nexel (2.5)**

*AO*, Arbeitsgemeinschaft für Osteosynthesefragen; *RCT*, randomized controlled trial; *NR*, not reported; *OTA*, Orthopaedic Trauma Association.

Bold text emphasizes the weighted outcomes.

∗ Total number of patients.

**Table II:** Baseline characteristics for articles reporting on elbow hemiarthroplasty.

Study	Patients, n	Mean follow-up, mo	Minimum follow-up, mo	Mean age, yr	Design	Fracture type (AO/OTA)	Days from injury to surgery	Implants used
Stephens^57^	9	44.1 (18.3)	23	71 (55-92)	Case-series	NR	NR	Tornier Latitude (n = 9)
Taylor^59^	7	29.9 (11.9)	11.4	72.1 (9.1)	Case-series	NR	NR	Tornier Latitude (n = 7)
Jonsson^25^	18	NR	24	74 (8.5)	RCT	B3 (n = 9) C1 (n = 8) C3 (n = 1)	7.7 (4.7)	Tornier Latitude (n = 18)
Nestorson^42^	42	34.3 (9.3)	24	72 (56-84)	Cohort study	B3 (n = 6) C2 (n = 17) C3 (n = 2) Multiplane (n = 17)	5.8 (0-15)	Tornier Latitude (n = 42)
Phadnis^44^	16	35 (15)	24	78.7 (60-90)	Case-series	B3 (n = 1) C2 (n = 2) C3 (n = 13)	13.3 (6.8)	Tornier Latitude (n = 16)
Burkhart^11^	8	12.1 (4.3)	6	75.2 (62-88)	Case-series	B3 (n = 3) C3 (n = 5)	NR	Tornier Latitude (n = 8)
Al-Hamdani^2^	24	20 (14.5)	12	65 (47-80)	Cohort study	C (n = 24)	NR	Tornier Latitude (n = 18)
Adolfsson^1^	8	50 (11.3)	25	79 (71-89)	Case-series	B3 (n = 3) C3 (n = 5)	NR	Kudo (n = 8)
Dirckx^15^	18	32 (9.5)	18	74.5 (7.9)	Cohort study	B3 (n = 2) C3 (n = 16)	NR	NR
Hohman^21^	5	36 (8.5)	26	68.4 (59-75)	Case-series	C3 (n = 5)	NR	Tornier Latitude (n = 5)
Jenkins^24^	37	61 (20.3)	24	75 (29-93)	Case-series	C3 (n = 37)	NR	Tornier Latitude (n = 37)
**Weighted**	**192**∗	**34.4 (23.4-45.3)**	**20.8 (17.5-24.1)**	**73.0 (70.5-75.4)**	**Case series (46.9%)** **Cohort study (43.7%)** **RCT (9.4%)**	**A (0.0%)** **B (15.9%)** **C (84.1%)**	**3.1 (0.9-5.3)**	**Tornier Latitude (95.7%)** **Kudo (4.3%)**

*AO*, Arbeitsgemeinschaft für Osteosynthesefragen; *RCT*, randomized controlled trial; *NR*, not reported; *OTA*, Orthopaedic Trauma Association.

Bold text emphasizes the weighted outcomes.

∗ Total number of patients.

### Primary outcome

**Figure 2:**
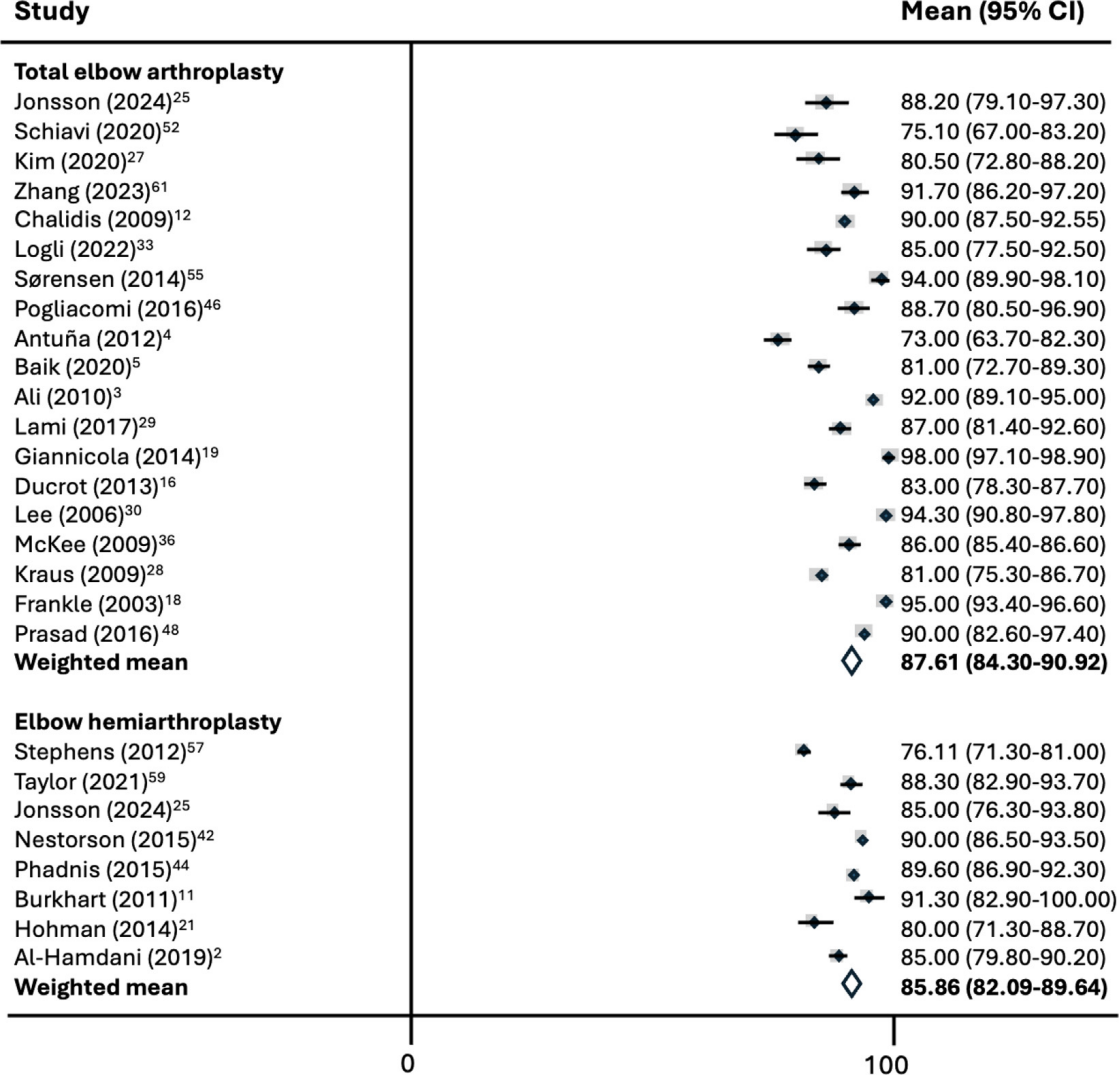
Meta-analysis of mean MEPS by implant type. *MEPS*, Mayo Elbow Performance Score; *CI*, confidence interval.

A total of 19 studies reported MEPS outcomes following TEA, yielding a weighted mean score of 87.6 (95% CI: 84.3-90.9; I^2^ = 96.9%) (Fig. 2). Eight studies assessed MEPS following EHA, reporting a weighted mean score of 85.9 (95% CI: 82.1-89.6; I^2^ = 76.8%). No statistically significant difference was detected between the groups (*P* = .505).

### Secondary outcomes

**Figure 3:**
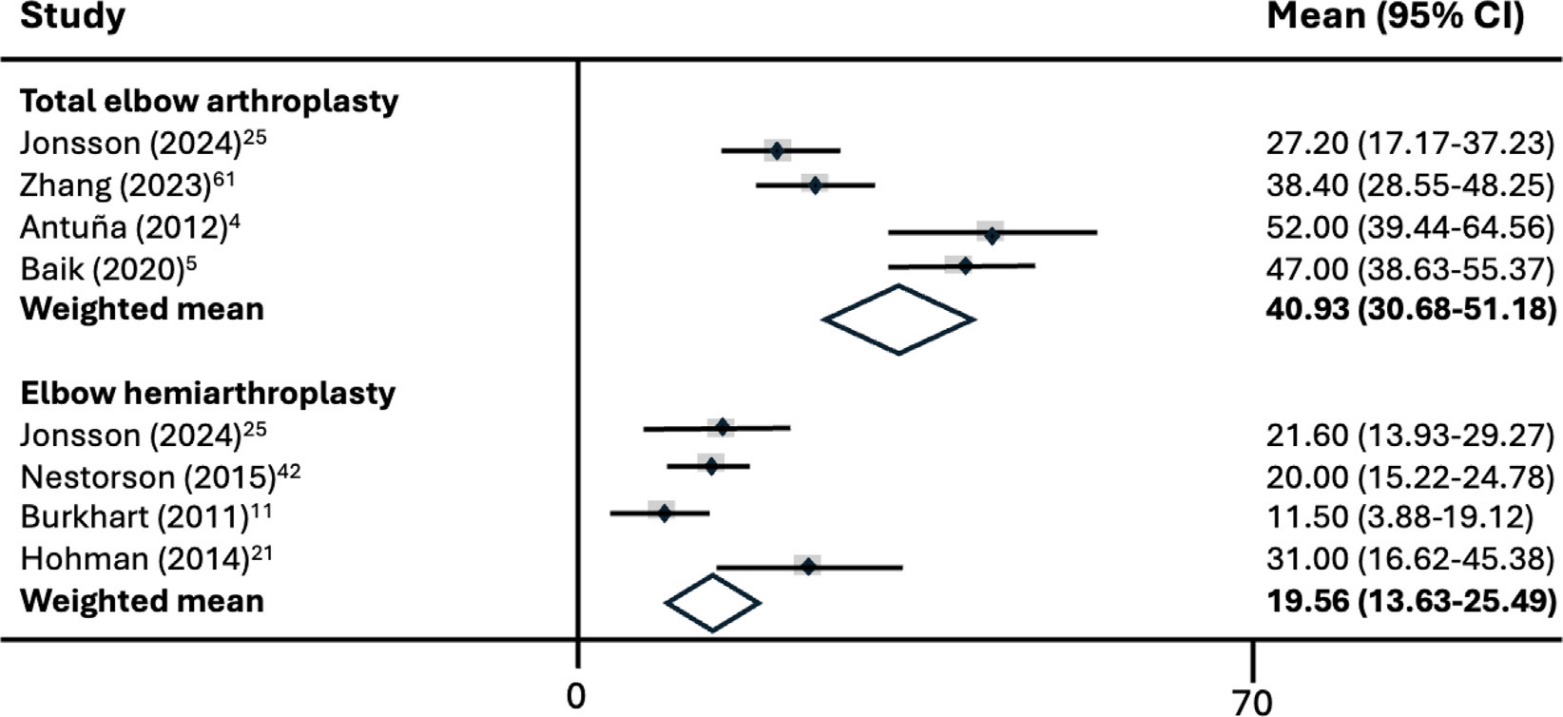
Meta-analysis of mean DASH score by implant type. *DASH*, Disabilities of the Arm, Shoulder and Hand; *CI*, confidence interval.

The DASH score was reported in four studies within the TEA group, resulting in a weighted mean of 40.9 (95% CI: 30.7-51.2; I^2^ = 75.9%), whereas the EHA group, based on four studies, had a weighted mean DASH score of 19.6 (95% CI: 13.6-25.5; I^2^ = 57.1%). This difference was statistically significant in favor of the EHA (*P* = .001), as can be seen in Figure 3.

**Figure 4:**
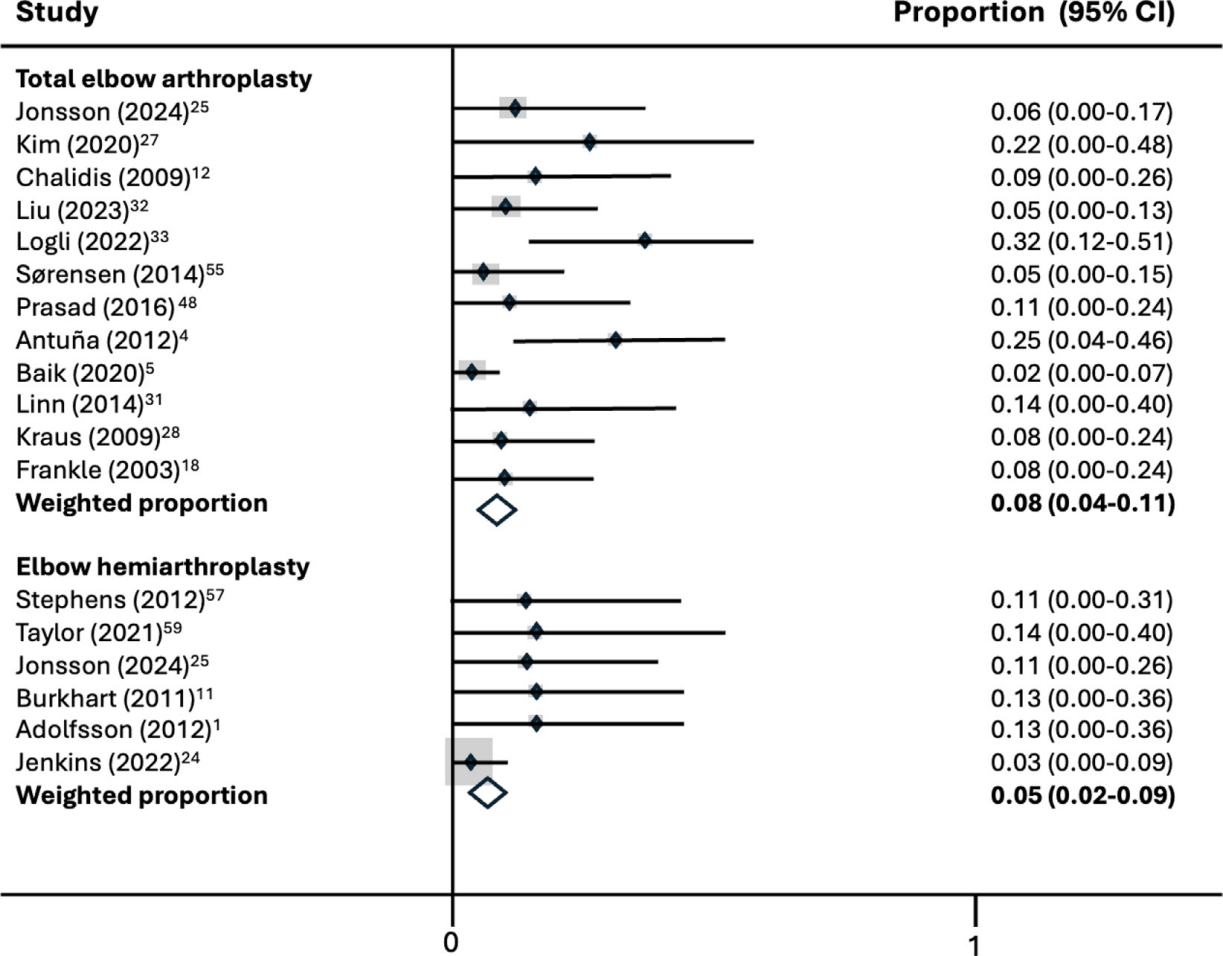
Meta-analysis of mean revision rate by implant type. *CI*, confidence interval.

Total implant-related complications were higher in the EHA group (11.1% vs. 7.6%). This, however, did not reach statistical significance and was mostly driven by the significantly higher rate of wear of the bushing elements in the EHA group (29.3% vs. 11.9%; *P* = .027). The reintervention rate was comparable for both TEA (6.4%) and EHA (3.3%; *P* = .051). All implant-related complications including whether they required reinterventions are listed in Table III. A meta-analysis of mean revision rate by implant type is presented in Figure 4.

**Table III:** Pooled risks of implant-related complications with relative risk.

Implant-related complication	Pooled risk TEA			Pooled risk EHA			RR (CI 95%)	*P* value
	Proportion	Percentage (%)	Studies	Proportion	Percentage (%)	Studies		
Total implant-related complications	47/621	7.6	26	34/305	11.1	11	0.68 (0.45-1.03)	.070
Revision surgery	40/621	6.4	26	10/305	3.3	11	1.96 (0.99-3.88)	.051
Periprosthetic fracture	16/198	8.0	7	3/33	9.0	3	0.89 (0.27-2.88)	.740
Revision surgery	16/198	8.0	7	2/33	6.0	3	1.33 (0.32-5.53)	1.000
Mechanical failure	6/98	6.1	6	1/15	6.7	4	0.92 (0.12-7.11)	.935
Revision surgery	6/98	6.1	6	1/15	6.7	4	0.92 (0.12-7.11)	.935
Aseptic loosening	13/172	7.6	9	3/50	6.0	2	1.26 (0.37-4.25)	1.000
Revision surgery	13/172	7.6	9	2/50	4.0	2	1.89 (0.44-8.10)	.530
Dislocation	1/12	8.3	1	4/62	6.5	4	1.29 (0.16-10.57)	1.000
Revision surgery	1/12	8.3	1	4/62	6.5	4	1.29 (0.16-10.57)	.515
Bushing wear	8/67	11.9	5	17/58	29.3	3	**0.41 (0.19-0.87)**	**.027**
Revision surgery	1/67	1.5	5	0/58	0	3	1.75 (0.06-51.11)	1.000
Epicondyle non-union	1/35	2.9	1	5/60	8.3	2	0.34 (0.04-2.82)	.291
Revision surgery	0/35	0	1	0/60	0	2	1.70 (0.03-84.0)	1.000
Prominent hardware	3/39	7.7	2	1/27	3.7	2	2.08 (0.23-18.92)	.639
Revision surgery	3/39	7.7	2	1/27	3.7	2	2.08 (0.23-18.92)	.639

*RR*, relative risk; *EHA*, elbow hemiarthroplasty; *TEA*, total elbow arthroplasty; *CI*, confidence interval.

Bolded text emphasizes the significant outcomes.

Total general complications were also comparable for TEA (9.5%) and the EHA group (11.6%; relative risk [RR] 0.81 [95% CI 0.61-1.07]; *P* = .145) with the most common complications being heterotopic ossification and postoperative ulnar neuropathy. The need for reintervention for general complications was equally low for both TEA (2.3%) and EHA (2.3%; *P* = .992). All general complications and the need for reinterventions are described in Table IV.

**Table IV:** Pooled risks of general postoperative complications with relative risk.

General postoperative complications	Pooled risk TEA			Pooled risk EHA			RR (CI 95%)	*P* value
	Proportion	Percentage (%)	Studies	Proportion	Percentage (%)	Studies		
Total general postoperative complications	114/1204	9.5	26	70/600	11.6	11	0.81 (0.61-1.07)	.145
Treatment	28/1204	2.3	26	14/600	2.3	11	1.00 (0.53-1.88)	.992
Deep infection	12/265	4.5	7	1/34	2.9	3	1.53 (0.21-11.47)	.674
DAIR	6/265	23.1	7	1/34	2.9	3	0.77 (0.10-6.20)	.574
Septic loosening	2/16	12.5	1	0/37	0	1	11.2 (0.6-220.5)	.112
Revision	2/16	12.5	1	0/37	0	1	11.2 (0.6-220.5)	.112
Superficial infection	7/137	5.1	4	1/58	1.7	4	2.96 (0.37-23.55)	.440
Antibiotics	1/137	5.1	4	0/58	0	4	1.28 (0.05-31.03)	.878
Wound dehiscence	10/169	5.9	7	1/37	2.7	1	2.19 (0.29-16.58)	.693
Aseptic closure	3/169	1.8	7	0/37	0	1	1.56 (0.08-29.67)	.766
Heterotopic ossification	26/93	28.0	16	36/127	28.3	5	0.99 (0.64-1.51)	.949
HO resection	7/93	7.5	16	4/127	3.1	5	2.39 (0.72-7.93)	.210
Stiffness	5/160	3.1	8	6/79	7.6	3	0.41 (0.13-1.31)	.186
Arthrolysis	3/160	1.9	8	5/79	6.3	3	0.30 (0.73-1.21)	.120
Joint instability	1/28	3.6	2	2/60	3.0	4	1.07 (0.10-11.33)	.954
Ligament repair	1/28	3.6	2	1/60	3.0	4	2.14 (0.14-33.03)	.538
Ulnar neuropathy	51/336	15.2	17	23/168	13.7	9	1.11 (0.70-1.75)	.656
Ulnar nerve transposition	5/336	1.5	17	3/168	1.8	9	0.83 (0.20-3.45)	.801

*RR*, relative risk; *EHA*, elbow hemiarthroplasty; *TEA*, total elbow arthroplasty; *CI*, confidence interval; *DAIR*, débridement, antibiotics, and implant retention; *HO*, heterotopic ossification.

In terms of ROM, the mean flexion in the TEA group was 128.2° (95% CI 127.4-129.0°), compared to 127.1° (95% CI 125.3-128.9°) in the EHA group (*P* = .274). The weighted mean extension deficit was 16.0° (95% CI 15.2-16.8°) for TEA and 20.2° (95% CI 18.4-22.1°) for EHA (*P* = .003). Mean supination in the TEA group was 81.0° (95% CI: 80.4-81.6°), whereas in the EHA group, it was 82.5° (95% CI: 80.9-84.0° [*P* = .077]). Mean pronation was 80.7° (95% CI: 79.8-81.5°) in the TEA group and 82.2° (95% CI: 80.9-83.6°) in the EHA group (*P* = .065). A detailed overview of ROM outcomes is provided in Supplementary S8-S11.

### Sensitivity analysis

Sensitivity analyses revealed no statistically significant differences in the EHA and TEA groups: pooled mean MEPS was 88.2 (85.4-91.1) among the high-quality studies vs. 86.3 (84.2-88.3) among the low-quality studies (*P =* .281) in the EHA group. In the TEA group the pooled mean MEPS was 85.5 (80.8-90.3) among the high-quality studies vs. 88.4 (83.1-93.6) among the low-quality studies (*P* = .374).

## Discussion

### Summary of results

This proportional meta-analysis included 37 studies with 694 patients—502 in the TEA group and 192 in the EHA group. The MEPS score was comparable between the two techniques (87.6 vs. 85.9; *P* = .505). Patients treated with an EHA had a better DASH score (19.6 vs. 40.9; *P* = .001). Wear occurred more frequently in patients with EHA (11.9% vs. 29.3%; *P* = .027). This, however, rarely required reintervention. All other complications and the overall need for reintervention were similar across treatment groups. Among ROM parameters, only extension deficit showed a significant difference, favoring TEA (16.0° vs. 20.2°; *P* = .002).

### Comparison to previous literature

To our knowledge, only one meta-analysis has been published so far that compares TEA and EHA for distal humerus fractures in elderly patients. The meta-analysis by Burden et al also examined the outcomes of EHA and TEA in patients over the age of 65 with unreconstructable distal humerus fractures.^10^ By extending the search period by five years and additionally including Jonsson et al's RCT and eight observational studies, this meta-analysis offers a substantial update.^25^ Moreover, the present meta-analysis gives a meta-analysis on a wider range of outcomes, not only complications but also of the MEPS and DASH scores. In addition, complications in this study were categorized into general postoperative and implant-related complications and were directly associated with their corresponding treatment interventions to clarify the clinical consequences of the complications.

Their findings were comparable to ours; the DASH score was better in the EHA group compared to the TEA group (19.6 [standard deviation (SD) 7.5] vs. 38.0 [SD 11.9]). However, there was no significant difference in the MEPS between the TEA and EHA groups (87.0 vs. 88.3) and no significant difference was found in the complication rate with reported rates of 22% (95% CI: 5-44%) and 21% (95% CI: 13-30%), respectively.

### Interpretation of results and clinical implications

To date, TEA has been the preferred implant for treating unreconstructable distal humerus fractures in the elderly. This preference is most likely caused by the fact that orthopedic surgeons prefer to use implants that they are most familiar with. Since TEA is commonly used for treating elbow arthrosis, the same orthopedic surgeon will be more inclined to use this implant for fractures as well.^51^ However, the findings of this study challenge that justification.

EHA seems to yield similar functional outcomes and ROM as TEA while also showing similar complication rates. A significant advantage of EHA is that it allows patients to fully weightbear, whereas TEA imposes strict lifting restrictions. This distinction explains why the MEPS, which focus on elbow-specific function, are comparable between both groups, whereas DASH scores, which reflect the overall upper extremity function, show notable differences.^23^^,^^39^ MEPS assesses activities like combing hair, personal hygiene, dressing, and eating—tasks that are generally unaffected by lifting restrictions. In contrast, DASH includes activities such as opening heavy doors, carrying objects, and performing household chores (eg, cleaning walls), which likely exceed typical lifting limitations.^9^ However, it should be noted that difficulties with these activities may also be influenced by other upper extremity conditions in the elderly, such as rotator cuff injuries or osteoarthritis.^41^^,^^60^

Although wear of the bushing elements is observed more frequently in the EHA groups—likely as a direct consequence of full weight-bearing—it rarely necessitates reintervention, raising questions about its clinical relevance. That said, this study examines an older patient population only. In this group, wear appears to have minimal clinical consequences, but younger patients with a longer life span may face more issues with wear over time.

Overall, elderly patients with unreconstructable distal humerus fractures seem to benefit more from EHA than TEA. Although transitioning from TEA to EHA may require a certain learning curve, most studies on EHA included in this meta-analysis were case series describing early experiences. This suggests that even in the initial stages of adoption EHA, similar outcomes can be expected as of the well-known TEA.

From a methodological perspective, this meta-analysis also challenges the routine exclusion of case series from meta-analyses due to the perceived high risk of confounding. Based on previous meta-analyses, observational studies in orthopedic trauma research seem less prone to confounding as previously conceived.^4^^,^^17^^,^^22^ The reason lies in the fact that many surgical treatment decisions in the orthopedic field (such as the choice on EHA or TEA) are based on preference and expertise of the treating surgeon rather than patient-specific risk factors. Consequently, confounding is less of a concern in this area compared to pharmacological research, where sicker patients often receive more advanced treatments, introducing potential bias. In the present meta-analysis, including RCTs, observational studies, and case-series, baseline characteristics were similar across treatment groups. This supports the claim that confounding is less of an issue for this particular research question (surgical treatment vs. surgical treatment) and the inclusion of case series in this meta-analysis seems justified.^6^^,^^7^

### Limitations

Several limitations must be acknowledged. Firstly, the inclusion of case series inevitably results in an overall level IV evidence grade for the review. However, unlike previous reviews, an RCT was included in the analysis. In addition, although statistical heterogeneity for the primary outcome (MEPS) was high, the absolute MEPS values showed only minor variation, with all results clustered between 80 and 90. This indicates that although heterogeneity may be statistically significant, it is likely of little clinical relevance. To explore study quality as a potential source of heterogeneity, a subgroup analysis was performed. The weighted mean of the top 50th percentile and the bottom 50th percentile showed no statistically significant differences in the mean MEPS within the TEA and EHA groups. Thus, we assume that the heterogeneity is not caused by the study quality and maintain the justification for including case series in this meta-analysis.

Secondly, the variability in the definition of complications across studies makes it questionable to aggregate all reported complications from different studies into a single summary. Differences in classification criteria, severity thresholds, and reporting standards can lead to inconsistencies, potentially distorting overall complication rates and limiting the reliability of pooled analyses.

Thirdly, no distinction was made between unlinked, semiconstrained and linked total elbow prostheses in the analysis, which may introduce variability in outcomes. These three designs have different biomechanical properties and failure patterns, making it important to analyze them separately to ensure accurate comparisons and conclusions. Although linked implants appear to have less instability and dislocation at first glance, not all of them can withstand the high mechanical stresses, which may ultimately lead to failure of the central axis and bushing.^53^ Among the included studies, the semiconstrained Coonrad–Morrey elbow (Zimmer Biomet, Warsaw, IN, USA) was by far the most used implant, whereas the fully linked Discovery (Enovis, Wilmington, DE, USA) and Latitude systems (Stryker Kalamazoo, MI, USA) were used less frequently. This imbalance in implant selection could further influence pooled outcome analyses; however, the distinction between unlinked and linked prostheses could not be made with the reported data.

## Conclusion

TEA and EHA provide similar outcomes regarding MEPS, revision rates, and complication rates in elderly patients. However, TEA is associated with worse DASH scores, likely due to lifelong weight-lifting restrictions. This may support a growing preference for elbow hemiarthroplasty in elderly patients with unreconstructable distal humerus fractures.

## Acknowledgment

This work was part of the activities of the Natural Experiments Study Group (www.next-studygroup.org).

## Disclaimers:

Funding: No funding was disclosed by the authors.

Conflicts of interest: The authors, their immediate families, and any research foundation with which they are affiliated have not received any financial payments or other benefits from any commercial entity related to the subject of this article.
