# Supplementary material for: The current role of elbow hemiarthroplasty compared to total arthroplasty in the treatment of distal humerus fractures in the elderly. Twice the trouble or half the hassle?
Source: JSES Rev Rep Tech. 2025 Jul 10;5(4):984–93. doi: 10.1016/j.xrrt.2025.06.015 (PMC12573455; doi:10.1016/j.xrrt.2025.06.015)
Supplement: Supplementary Tables [file mmc1.docx]

**Concept 1: distal humerus fractures**

*Search query:* "Humeral Fractures, Distal"[Mesh] OR “distal humer* fractur*”[tiab] OR “elbow fractur*”[tiab] OR “elbow joint*”[tiab]

**Concept 2: total elbow arthroplasty**

*Search query*: “Arthroplasty, Replacement, Elbow"[Mesh] OR “total elbow arthroplast*”[tiab] OR “total elbow replacement*”[tiab]

**Concept 3: elbow hemi-arthroplasty**

*Search query:* “hemiarthroplast*”[tiab] OR “hemi-arthroplast*”[tiab] OR “humer* replacement*”[tiab] OR “hemi arthroplast*”[tiab]

**Concept 4: elderly**

*Search query*: (("Aged"[Mesh] OR "Aged"[Tiab] OR "Elderly"[Tiab]) OR ("Aged, 80 and over"[Mesh] OR "Frail Elderly"[Mesh]))

**Total search query:** (#1 AND #2 AND #4) OR (#1 AND #3 AND #4) OR (#1 AND #2 AND #3 AND #4)

Supplementary 1: PubMed search strategy.


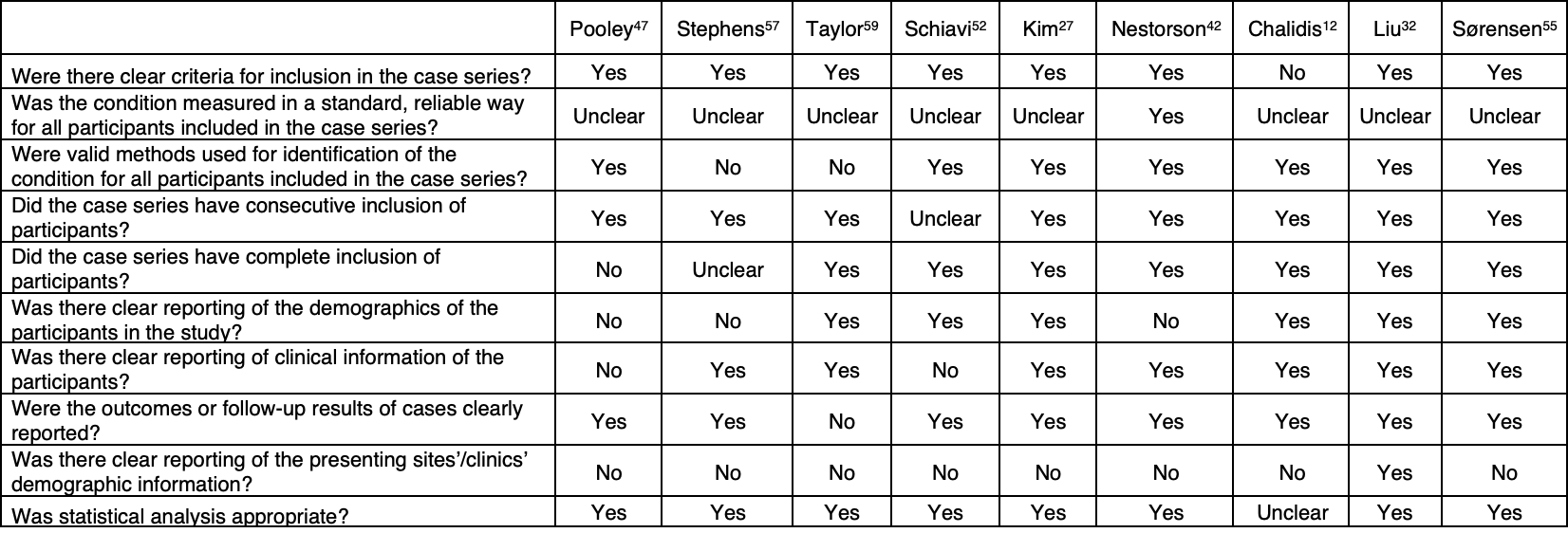


Supplementary 2.1: Quality assessment of case series using the Joanna Briggs Institute Critical Appraisal Tool (JBI).


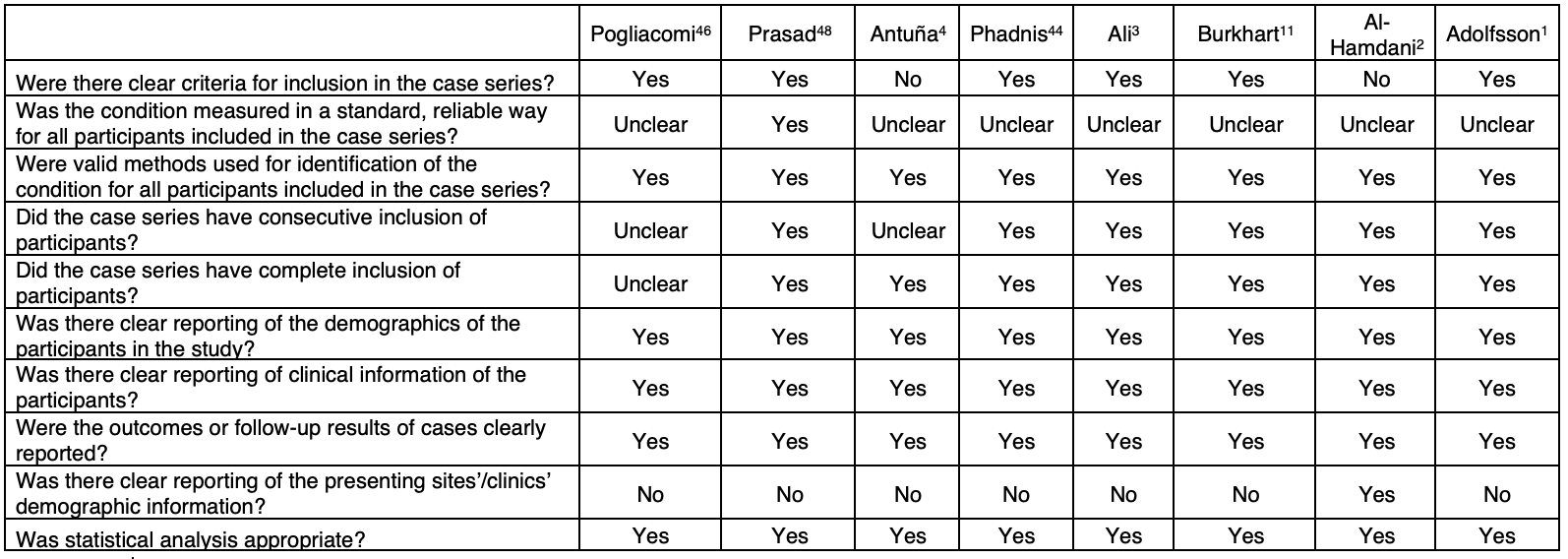


Supplementary 2.2: Quality assessment of case series using the Joanna Briggs Institute Critical Appraisal Tool (JBI).


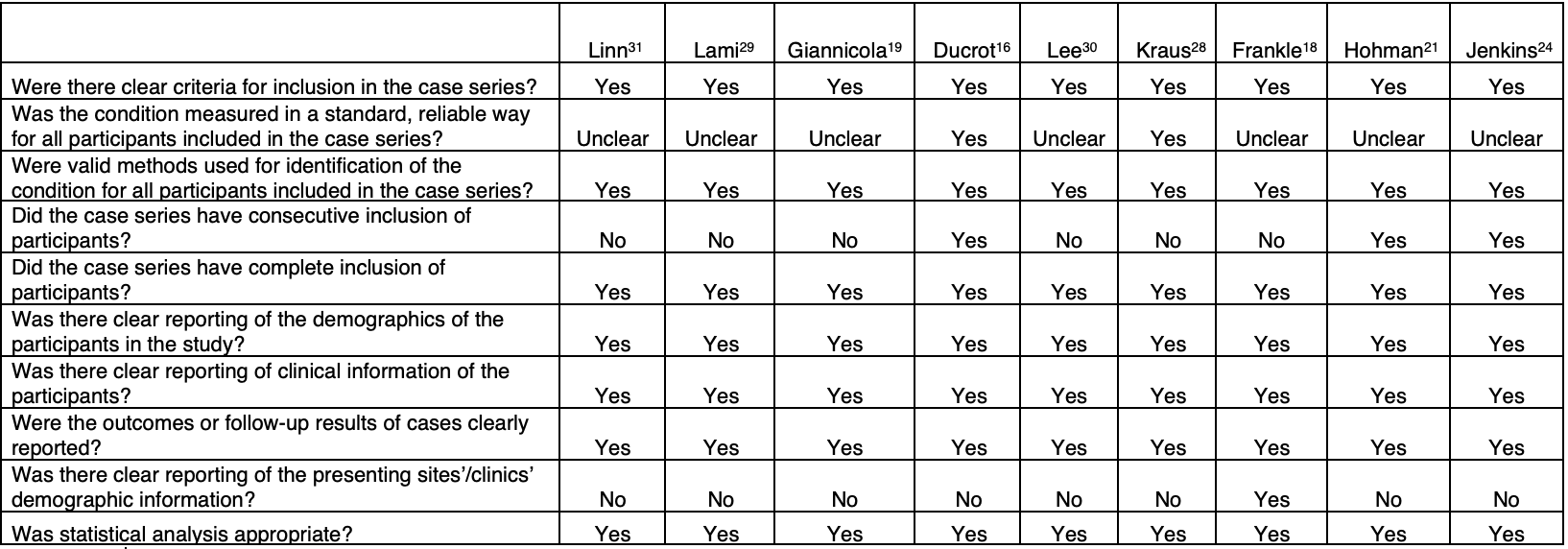


Supplementary 2.3: Quality assessment of case series using the Joanna Briggs Institute Critical Appraisal Tool (JBI).


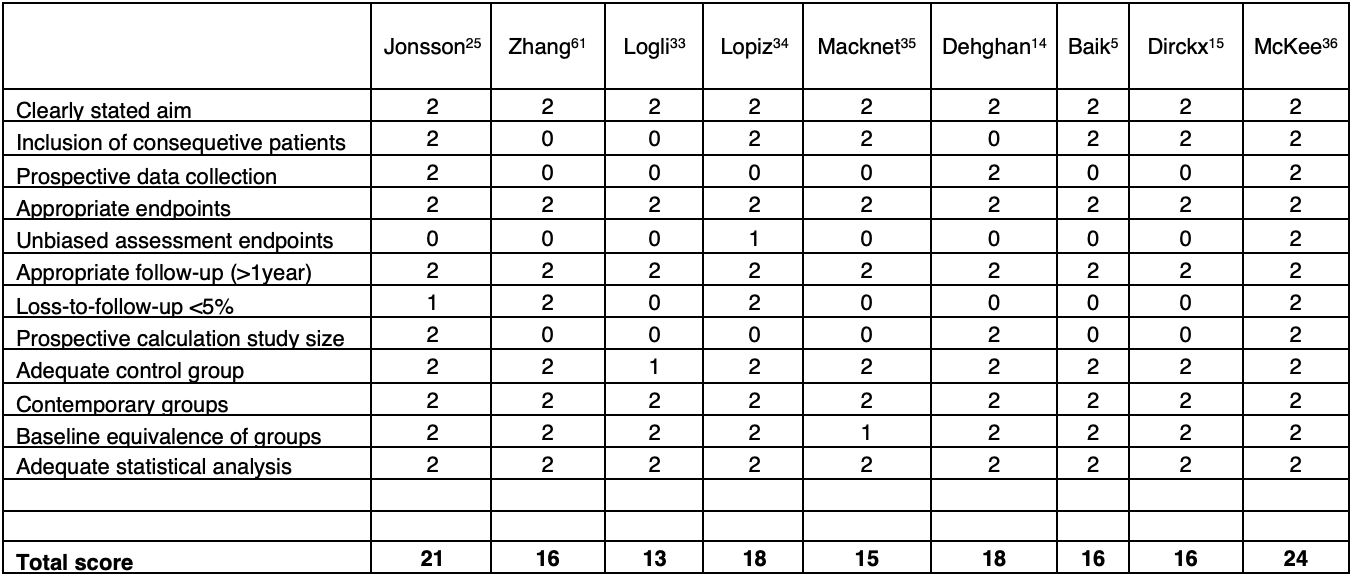


Supplementary 3: Quality assessment of observational comparative studies using the Methodological Index for Non-Randomized Studies (MINORS) tool.

| **Domains** | **Risk-of-bias judgement** |
| --- | --- |
| D1: Randomization | Low |
| D2: Intervention | Some concerns |
| D3: Missing data | Low |
| D4: Measurement | Low |
| D5: Selection | Some concerns |
| Overall | Some concerns |

Supplementary 4: Quality assessment of the one randomized controlled trial (Jonsson 2024**)** using the Cochrane Risk of Bias Tool (RoB-2).

| **Study** | **Rank** | **Quality top 50^th^ percentile** |
| --- | --- | --- |
| Stephens^57^ | 9 | 0 |
| Taylor^59^ | 8 | 0 |
| Jonsson^21^ | 1 | 1 |
| Nestorson^53^ | 4 | 1 |
| Phadnis^54^ | 7 | 0 |
| Burkhart^55^ | 2 | 1 |
| Al-Hamdani^2^ | 5 | 0 |
| Adolfsson^57^ | 6 | 0 |
| Hohman^59^ | 3 | 1 |

Supplementary 5.1: Ranking of studies reporting the MEPS for EHA based on study assessment.

| **Study** | **Rank** | **Quality top 50^th^ percentile** |
| --- | --- | --- |
| Jonsson^21^ | 1 | 1 |
| Schiavi^52^ | 9 | 1 |
| Kim^27^ | 8 | 1 |
| Zhang^29^ | 11 | 0 |
| Chalidis^12^ | 17 | 0 |
| Logli^33^ | 13 | 0 |
| Sørensen^55^ | 7 | 1 |
| Pogliacomi^46^ | 16 | 0 |
| Prasad^48^ | 5 | 1 |
| Antuña^4^ | 17 | 0 |
| Baik^5^ | 12 | 0 |
| Ali^3^ | 4 | 1 |
| Lami^29^ | 14 | 0 |
| Giannicola^19^ | 15 | 0 |
| Ducrot^16^ | 3 | 1 |
| Lee^30^ | 18 | 0 |
| McKee^36^ | 2 | 1 |
| Kraus^28^ | 6 | 1 |
| Frankle ^18^ | 10 | 0 |

Supplementary 5.2: Ranking of studies reporting the MEPS for TEA based on study assessment.

| **Study** | **Mean MEPS** | **SD** | **Quality top half** | **n total** |
| --- | --- | --- | --- | --- |
| Stephens^57^ | 76.11 | 6.3 | 0 | 9 |
| Taylor^59^ | 88.3 | 5.8 | 0 | 7 |
| Jonsson^21^ | 85 | 17.6 | 1 | 18 |
| Nestorson^53^ | 90 | 11.2 | 1 | 42 |
| Phadnis^54^ | 89.6 | 5 | 0 | 16 |
| Burkhart^55^ | 91.3 | 10 | 1 | 8 |
| Al-Hamdani^2^ | 85 | 12.5 | 0 | 24 |
| Adolfsson^57^ | 93 | 3.44 | 0 | 8 |
| Hohman^59^ | 80 | 7 | 1 | 5 |
| **Pooled mean MEPS high-quality** | **88.2 (85.4-91.1)** | ***p =* 0.281** | | |
| **Pooled mean MEPS low-quality** | **86.3 (84.2-88.3)** |  |  |  |

Supplementary 6: Sensitivity analysis on the MEPS for EHA studies.

| **Study** | **Mean MEPS** | **SD** | **Quality top half** | **n total** |
| --- | --- | --- | --- | --- |
| Jonsson^21^ | 88.2 | 17.7 | 1 | 17 |
| Schiavi^52^ | 75.1 | 12.8 | 1 | 12 |
| Kim^27^ | 80.5 | 10 | 1 | 9 |
| Zhang^29^ | 91.7 | 6 | 0 | 13 |
| Chalidis^12^ | 90 | 3.8 | 0 | 11 |
| Logli^33^ | 85 | 17 | 0 | 22 |
| Sørensen^55^ | 94 | 8.8 | 1 | 20 |
| Pogliacomi^46^ | 88.7 | 17.5 | 0 | 20 |
| Prasad^48^ | 90 | 13.0 | 1 | 19 |
| Antuña^4^ | 73 | 17.5 | 0 | 16 |
| Baik^5^ | 81 | 27 | 0 | 43 |
| Ali^3^ | 92 | 6.3 | 1 | 20 |
| Lami^29^ | 87 | 12.3 | 0 | 21 |
| Giannicola^19^ | 98 | 1.3 | 0 | 10 |
| Ducrot^16^ | 83 | 10 | 1 | 20 |
| Lee^30^ | 94.3 | 3.8 | 0 | 7 |
| McKee^36^ | 86 | 1.5 | 1 | 25 |
| Kraus^28^ | 81 | 9 | 1 | 12 |
| Frankle ^18^ | 95 | 2.5 | 0 | 12 |
| **Pooled mean MEPS high-quality** | **85.5 (80.8-90.3)** | ***p* = 0.374** | | |
| **Pooled mean MEPS low-quality** | **88.4 (83.1-93.6)** |  |  |  |

Supplementary 7: Sensitivity analysis on the MEPS for TEA studies.

| **TEA** | |  | |  |  |  |
| --- | --- | --- | --- | --- | --- | --- |
|  | |  | |  |  |  |
| **Study** | | **Sample size** | | **Mean** | **SD** | **CI 95%** |
| Jonsson^21^ | | 17 | | 136 | 11 | 130.7-141.2 |
| Kim^28^ | | 9 | | 127.7 | 11.3 | 120.3-135.1 |
| Chalidis^30^ | | 11 | | 117 | 7.5 | 112.6-121.4 |
| Liu^31^ | | 22 | | 129.4 | 11.5 | 124.6-134.2 |
| Logli^32^ | | 22 | | 123 | 22 | 113.8-132.2 |
| Lopiz^33^ | | 11 | | 117 | 9.6 | 111.3-122.7 |
| Sørensen^34^ | | 20 | | 114 | 15 | 107.4-120.6 |
| Macknet^38^ | | 69 | | 130 | 5 | 128.8-131.2 |
| Antuña^39^ | | 16 | | 117 | 30 | 102.3-131.7 |
| Baik^41^ | | 43 | | 124 | 15 | 120.0-128.5 |
| Linn^43^ | | 7 | | 111 | 10 | 103.6-118.4 |
| Lami^44^ | | 21 | | 126 | 8.8 | 122.2-129.8 |
| Giannicola^45^ | | 10 | | 137.5 | 5 | 134.4-140.6 |
| Ducrot^46^ | | 20 | | 130 | 10.3 | 125.5-134.5 |
| Lee^47^ | | 7 | | 130 | 5 | 126.3-133.7 |
| McKee^48^ | | 25 | | 133 | 16.3 | 126.6-139.4 |
| Kraus^49^ | | 12 | | 120 | 7.5 | 115.8-124.2 |
| Frankle^50^ | | 12 | | 125 | 5 | 122.2-127.8 |
| **Weighted mean (CI 95%)** | | | | **128.22** |  | **127.4-129.0** |
|  | |  |  |  |  |  |
|  |  |  |  |  |  |  |
| **EHA** | |  | |  |  |  |
|  | |  | |  |  |  |
| **Study** | | **Sample size** | | **Mean** | **SD** | **CI 95%** |
| Taylor^52^ | | 7 | | 135 | 9 | 128.3-141.7 |
| Jonsson^21^ | | 18 | | 126 | 15 | 119.1-132.9 |
| Nestorson^53^ | | 42 | | 126.8 | 13.8 | 122.6-131.0 |
| Phadnis^54^ | | 16 | | 116 | 14 | 109.1-122.9 |
| Burkhart^55^ | | 8 | | 124.5 | 11.3 | 116.7-132.3 |
| Adolfsson^57^ | | 8 | | 127.5 | 5 | 124.0-131.0 |
| Dirckx^58^ | | 18 | | 120 | 20 | 110.8-129.2 |
| Hohman^59^ | | 5 | | 122 | 7,5 | 115.4-128.6 |
| Jenkins^60^ | | 37 | | 132 | 12.5 | 128.0-136.0 |
| **Weighted mean (CI 95%)** | | | | **127.08** |  | **125.3-128.9** |

Supplementary 8: Mean range of flexion. SD, standard deviation; CI, confidence interval.

| **TEA** |  |  |  |  |
| --- | --- | --- | --- | --- |
|  |  |  |  |  |
| **Study** | **Sample size** | **Mean** | **SD** | **CI 95%** |
| Jonsson^21^ | 17 | 29 | 18 | 20.4-37.6 |
| Kim^28^ | 9 | 13,8 | 11,3 | 6.4-21.2 |
| Chalidis^30^ | 11 | 10 | 7,5 | 5.6-14.4 |
| Liu^31^ | 22 | 16,9 | 19,6 | 8.7-25.1 |
| Lopiz^32^ | 11 | 38 | 17 | 28.0-48.0 |
| Antuña^39^ | 16 | 28 | 22,5 | 17.0-39.0 |
| Baik^40^ | 43 | 15 | 3 | 14.1-15.9 |
| Linn^43^ | 7 | 19,3 | 6,3 | 14.6-24.0 |
| Lami^44^ | 21 | 22 | 17,5 | 14.5-29.5 |
| Giannicola^45^ | 10 | 15 | 7,5 | 10.4-19.6 |
| Ducrot^46^ | 20 | 33 | 20 | 24.2-41.8 |
| Lee^47^ | 7 | 41,4 | 15 | 30.3-52.5 |
| McKee^48^ | 25 | 26 | 18 | 18.9-33.1 |
| Kraus^49^ | 12 | 33 | 13,8 | 25.2-40.8 |
| Frankle^50^ | 12 | 15 | 7,5 | 10.8-19.2 |
| **Weighted mean (CI 95%)** | | **15.99** |  | **15.19-16.78** |
|  |  |  |  |  |
|  |  |  |  |  |
| **EHA** |  |  |  |  |
|  |  |  |  |  |
| **Study** | **Sample size** | **Mean** | **SD** | **CI 95%** |
| Taylor^52^ | 7 | 21 | 15 | 9.9-32.1 |
| Jonsson^21^ | 18 | 29 | 12 | 23.5-34.5 |
| Nestorson^53^ | 42 | 23.5 | 15 | 19.0-28.0 |
| Burkhart^55^ | 8 | 17.5 | 6,3 | 13.1-21.9 |
| Adolfsson^57^ | 8 | 31 | 10 | 24.1-37.9 |
| Dirckx^58^ | 18 | 10 | 10 | 5.4-14.6 |
| Hohman^59^ | 5 | 21 | 7,5 | 14.4-27.6 |
| Jenkins^60^ | 37 | 19 | 12,5 | 15.0-23.0 |
| **Weighted mean (CI 95%)** | | **20.21** |  | **18.37-22.05** |

Supplementary 9: Mean range of extension. SD, standard deviation; CI, confidence interval.

| **TEA** |  |  |  |  |
| --- | --- | --- | --- | --- |
|  |  |  |  |  |
| **Study** | **Sample size** | **Mean** | **SD** | **CI 95%** |
| Jonsson^21^ | 17 | 75 | 14 | 38.34-81.66 |
| Kim^28^ | 9 | 74,4 | 2,5 | 72.77-76.03 |
| Chalidis^30^ | 11 | 61 | 6,3 | 57.28-64.72 |
| Liu^31^ | 22 | 77,4 | 22,6 | 67.96-86.84 |
| Logli^32^ | 22 | 75 | 22 | 65.81-84.19 |
| Lopiz^33^ | 11 | 75 | 4 | 72.64-77.36 |
| Antuña^39^ | 16 | 75 | 22,5 | 63.98-86.03 |
| Baik^41^ | 43 | 84 | 6 | 82.21-85.79 |
| Ali^42^ | 20 | 77 | 12,5 | 71.52-82.48 |
| Giannicola^45^ | 10 | 84 | 1,3 | 83.19-84.81 |
| Lee^47^ | 7 | 72,9 | 10 | 65.49-80.31 |
| Kraus^49^ | 12 | 80 | 12,5 | 72.92-87.07 |
| **Weighted mean (CI 95%)** | | **80.99** |  | **80.37-81.61** |
|  |  |  |  |  |
|  |  |  |  |  |
| **EHA** |  |  |  |  |
|  |  |  |  |  |
| **Study** | **Sample size** | **Mean** | **SD** | **CI 95%** |
| Taylor^52^ | 7 | 84 | 8 | 78.08-89.93 |
| Jonsson^21^ | 18 | 81 | 9 | 76.84-85.16 |
| Phadnis^54^ | 16 | 85 | 5 | 82.55-87.45 |
| Burkhart^55^ | 8 | 79.5 | 10 | 72.57-86.43 |
| Hohman^59^ | 5 | 77 | 6,3 | 71.48-82.52 |
| Jenkins^60^ | 37 | 81 | 10 | 77.78-84.22 |
| **Weighted mean (CI 95%)** | | **82.46** |  | **80.89-84.04** |

Supplementary 10: Mean range of supination. SD, standard deviation; CI, confidence interval.

| **TEA** |  |  |  |  |
| --- | --- | --- | --- | --- |
|  |  |  |  |  |
| **Study** | **Sample size** | **Mean** | **SD** | **CI 95%** |
| Jonsson^21^ | 17 | 74 | 10 | 69.25-78.75 |
| Kim^29^ | 9 | 73,3 | 5 | 70.03-76.57 |
| Chalidis^30^ | 11 | 60 | 6,3 | 56.28-63.72 |
| Liu^31^ | 22 | 77,1 | 20,5 | 68.53-85.67 |
| Logli^32^ | 22 | 82 | 16 | 75.31-88.69 |
| Lopiz^33^ | 11 | 75 | 5 | 72.04-77.95 |
| Antuña^39^ | 16 | 78 | 7,5 | 74.33-81.68 |
| Baik^41^ | 43 | 89 | 6 | 87.21-90.79 |
| Ali^42^ | 20 | 74 | 12,5 | 68.52-79.48 |
| Giannicola^45^ | 10 | 83 | 2,5 | 81.45-84.55 |
| Lee^47^ | 7 | 75,7 | 10 | 68.29-83.11 |
| Kraus^49^ | 12 | 82 | 7,5 | 77.76-86.24 |
| **Weighted mean (CI 95%)** | | **80.65** |  | **79.77-81.54** |
|  |  |  |  |  |
|  |  |  |  |  |
|  |  |  |  |  |
| **EHA** |  |  |  |  |
|  |  |  |  |  |
| **Study** | **Sample size** | **Mean** | **SD** | **CI 95%** |
| Taylor^52^ | 7 | 87 | 5 | 83.30-90.70 |
| Jonsson^21^ | 18 | 78 | 5 | 75.69-80.31 |
| Phadnis^54^ | 16 | 85 | 5 | 82.55-87.45 |
| Burkhart^55^ | 8 | 80.5 | 7,5 | 75.30-85.70 |
| Hohman^59^ | 5 | 90 | 5 | 85.62-94.38 |
| Jenkins^60^ | 37 | 74 | 15 | 69.17-78.83 |
| **Weighted mean (CI 95%)** | | **82.24** |  | **80.90-83.58** |

Supplementary 11: Mean range of pronation. SD, standard deviation; CI, confidence interval.
